# Reducing Medication‐Related Harm Through Home‐Based Primary Care

**DOI:** 10.1111/jgs.70292

**Published:** 2025-12-31

**Authors:** Thomas J. Haferkamp, Nora Spadoni, Lauren J. Gleason

**Affiliations:** ^1^ Department of Medicine, Section of Geriatrics and Palliative Medicine The University of Chicago Medicine Chicago Illinois USA; ^2^ The University of Chicago Pritzker School of Medicine Chicago Illinois USA

**Keywords:** home‐based primary care, interprofessional, polypharmacy, social determinants of health

## Abstract

This figure illustrates teaching strategies for the five domains of geriatric care, represented as the “5 M's”: Medication, Mentation, Mobility, Multi‐Complexity, and Matters Most. For each domain, Ms. T's clinical problems are listed with corresponding interventions.

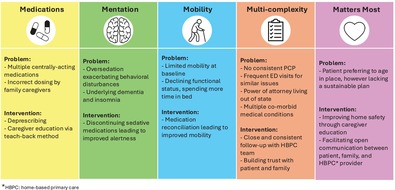

Ms. T is an 86‐year‐old Black woman with a history of Alzheimer's dementia, hypertension, hyperlipidemia, transient ischemic attack, gastroesophageal reflux disease, and anxiety who was referred to home‐based primary care (HBPC) following recurrent ED visits and hospitalizations for chest pain, anxiety, and shortness of breath. She was homebound and had not seen her primary care physician in 5 years. Ms. T's home medication regimen included paroxetine, amlodipine, atorvastatin, melatonin, pantoprazole. Following ED visits for agitation and insomnia, she was also prescribed quetiapine, mirtazapine, and trazodone. During her initial home visit, she described feeling “groggy, fatigued, and foggy.” Review of her medications revealed duplicate pill bottles and inconsistently‐filled pill boxes, highlighting challenges with medication management Figure 1. The HBPC physician organized her pill bottles in a designated location and recommended that her nephew, who is her primary caregiver, oversee her medications.

**FIGURE 1:**
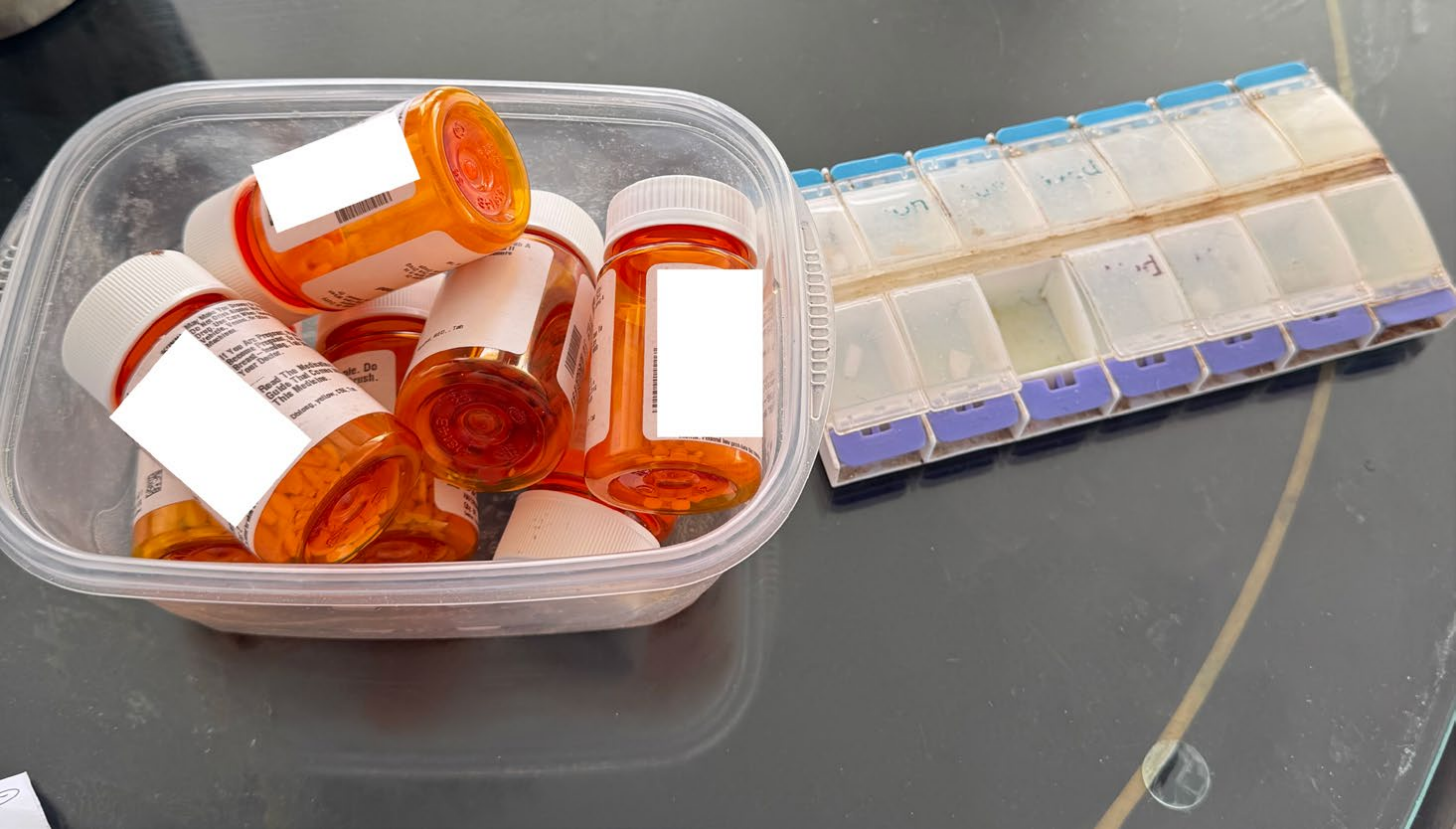
Ms. T's medications as they were initially presented to the HBPC team. These included duplicate medications and various fill dates. The pill set had different pills on different days without consistency.

Between subsequent home visits, episodes of chest pain and anxiety resulted in further ED presentations. Critically, her family accidentally administered double the quetiapine dose, which led to oversedation and a decline in personal hygiene. The HBPC team, including a pharmacist, nurse, and physician, worked with her family to consolidate her medications and discontinue unnecessary agents including mirtazapine and trazodone. They also provided education to her nephew on how to properly administer medications.

Ms. T's care was complicated by multiple medical issues and reliance on family caregivers, with her healthcare power of attorney (POA) living out of state. To address these challenges, the HBPC team emphasized clear communication when providing caregiver education. This included hands‐on medication teaching and providing regular home visits for close medication titration and monitoring.

Within a month, Ms. T demonstrated notable improvements in mobility and self‐care. She became more alert, resumed bathing, and independently answered the door for her care team. After 12 months of HBPC visits, Ms. T experienced no further ED visits or hospitalizations.

## 1 Main Teaching Points

**FIGURE 2:**
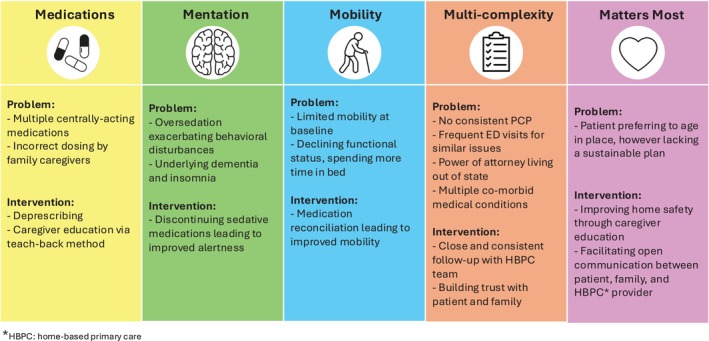
This figure illustrates teaching strategies for the five domains of geriatric care, represented as the “5 M's”: Medication, Mentation, Mobility, Multi‐Complexity, and Matters Most. For each domain, Ms. T's clinical problems are listed with corresponding interventions.

This case demonstrates how the 5 M's are often intertwined and interrelated. Polypharmacy (**Medications**) can have deleterious effects on older adults' **Mobility** and **Mentation**, particularly in patients with dementia. Inappropriate or incorrectly dosed medications can exacerbate functional decline, precipitate delirium, and lead to increased ED visits. Providing close follow‐up for older adults with dementia and underlying co‐morbid medical conditions (**Multi‐Complexity**) is a crucial part of re‐centering patient preferences (**Matters Most**). Continuity of primary care visits through a HBPC model allows for close titration of medications, optimization of functional and mental status, reduction of hospital utilization, and open communication with patient and family [1, 2, 3] Figure 2.

## 2 Discussion

Polypharmacy is a well‐known risk factor for adverse events in older adults, especially those with dementia [4]. Ms. T was prescribed multiple centrally‐acting medications, and incorrect dosing by family led to oversedation, worsening confusion, and declining personal hygiene and mobility—all of which limited her independence and ability to age in place. Ms. T's dementia also impacted her ability to communicate her symptoms and follow care recommendations. Social determinants of health (SDOH), including limited primary care follow‐up, reliance on family, out‐of‐state POA, and overall social isolation, further increased Ms. T's vulnerability to medication errors and poor health outcomes [5].

This case also demonstrates the need for effective caregiver education in order to reduce medication errors and improve patient outcomes [6]. The teach‐back method was utilized with Ms. T's nephew, who took over managing her medications, to ensure compliance and safe administration. This approach involves explaining medication regimens in clear terms, then asking the patient or caregiver to restate the information in their own words, allowing clinicians to address any misunderstandings. The teach‐back method has shown a positive impact in terms of patient satisfaction, readmission rates, and disease self‐management [7].

After close medication reconciliation and titration, as well as discontinuation of some centrally acting medications, Ms. T's hygiene, mobility, and overall function improved. This process, known as deprescribing, has been shown to reduce drug‐related adverse outcomes and rates of hospitalization in older adults [8]. The HBPC team played a central role in this case, allowing for close follow‐up, medication review, and real‐time adjustments based on Ms. T's progress. HBPC provides older adults with access to comprehensive, consistent, and patient‐centered care. These programs can reduce hospitalizations, improve patient quality of life, and lessen caregiver burden [9, 10]. Deprescribing efforts, improved caregiver engagement, and longitudinal care through the HBPC program helped honor Ms. T's wish to age in place and avoid facility placement.

The next steps in Ms. T's care involved practical, patient‐centered strategies to ensure continued stability and address polypharmacy. Regularly scheduled follow‐up visits were planned, with dedicated time for medication review at each appointment, allowing the HBPC team to further deprescribe and monitor for adverse effects. These interventions connect directly to the 5Ms by reducing unnecessary medications, supporting Ms. T's daily functioning, addressing her multiple healthcare needs, and centering care on what mattered most to her and her family. By simplifying medication management and involving family, the HBPC program addressed broader SDOH, ensuring sustainable and safe home‐based care for Ms. T.

## Author Contributions

Study concept and design: L.J.G., T.J.H. Drafting of the manuscript: T.J.H., N.S., and L.J.G. Critical revision of the manuscript for important intellectual content: T.J.H., N.S., and L.J.G.

## Funding

This work was supported by Health Resources and Services Administration, Geriatric Workforce Enhancement Program, U1QHP53056.

## Conflicts of Interest

The authors declare no conflicts of interest.
